# CD4 Enumeration Technologies: A Systematic Review of Test Performance for Determining Eligibility for Antiretroviral Therapy

**DOI:** 10.1371/journal.pone.0115019

**Published:** 2015-03-19

**Authors:** Rosanna W. Peeling, Kimberly A. Sollis, Sarah Glover, Suzanne M. Crowe, Alan L. Landay, Ben Cheng, David Barnett, Thomas N. Denny, Thomas J. Spira, Wendy S. Stevens, Siobhan Crowley, Shaffiq Essajee, Marco Vitoria, Nathan Ford

**Affiliations:** 1 London School of Hygiene and Tropical Medicine, London, WC1E 7HT, England; 2 Centre for Biomedical Research, Burnet Institute, Melbourne, 3004, Victoria, Australia; 3 Department of Immunology/Microbiology, Rush University Medical Center, Chicago, IL, 60612, United States of America; 4 Pangaea Global AIDS Foundation, Oakland, CA, 94607, United States of America; 5 UK NEQAS for Leucocyte Immunophenotyping, Sheffield, S10 2QD, England; 6 Duke Human Vaccine Institute and Center for HIV/AIDS, Immunology and Virology Quality Assessment Center, Durham, NC, 27710, United States of America; 7 Division of AIDS, STD, &TB Laboratory Research, National Center for Infectious Diseases, Centers for Disease Control and Prevention, Atlanta, GA, 30333, United States of America; 8 University of the Witwatersrand, Parktown, 2193, South Africa; 9 Director Health Programs, ELMA Philanthropies, New York, NY, United States of America; 10 Clinton Health Access Initiative, Boston, MA, 02127, United States of America; 11 World Health Organization, Geneva, Switzerland; University of Ottawa, CANADA

## Abstract

**Background:**

Measurement of CD4^+^ T-lymphocytes (CD4) is a crucial parameter in the management of HIV patients, particularly in determining eligibility to initiate antiretroviral treatment (ART). A number of technologies exist for CD4 enumeration, with considerable variation in cost, complexity, and operational requirements. We conducted a systematic review of the performance of technologies for CD4 enumeration.

**Methods and Findings:**

Studies were identified by searching electronic databases MEDLINE and EMBASE using a pre-defined search strategy. Data on test accuracy and precision included bias and limits of agreement with a reference standard, and misclassification probabilities around CD4 thresholds of 200 and 350 cells/μl over a clinically relevant range. The secondary outcome measure was test imprecision, expressed as % coefficient of variation. Thirty-two studies evaluating 15 CD4 technologies were included, of which less than half presented data on bias and misclassification compared to the same reference technology. At CD4 counts <350 cells/μl, bias ranged from -35.2 to +13.1 cells/μl while at counts >350 cells/μl, bias ranged from -70.7 to +47 cells/μl, compared to the BD FACSCount as a reference technology. Misclassification around the threshold of 350 cells/μl ranged from 1-29% for upward classification, resulting in under-treatment, and 7-68% for downward classification resulting in overtreatment. Less than half of these studies reported within laboratory precision or reproducibility of the CD4 values obtained.

**Conclusions:**

A wide range of bias and percent misclassification around treatment thresholds were reported on the CD4 enumeration technologies included in this review, with few studies reporting assay precision. The lack of standardised methodology on test evaluation, including the use of different reference standards, is a barrier to assessing relative assay performance and could hinder the introduction of new point-of-care assays in countries where they are most needed.

## Introduction

The increased availability of antiretroviral therapy (ART) has resulted in major reductions in morbidity and mortality in high HIV burden settings. Through significant global scale-up, access to ART is increasing, with around 10 million people in low- and middle-income settings receiving treatment as of the end of 2013, an estimated 65% of the of the global target of 15 million people set for 2015 [1].

CD4^+^ T-lymphocytes, also known as the helper T-cells, are the coordinators of the immune response that protects the body against microbial disease, a variety of autoimmune diseases and some forms of cancer. The destruction of CD4^+^ T-lymphocytes by HIV is the main cause of the progressive weakening of the immune system in HIV infection, and leads ultimately to acquired immune deficiency syndrome (AIDS). The CD4 count is a strong predictor of HIV progression to AIDS and death, and is considered the best laboratory marker for deciding when to initiate ART [2,3]. The use of clinical staging alone to determine the timing of ART initiation is limited by the unreliable correlation between asymptomatic or mild disease and short-term prognosis and may result in dangerous delays in treatment initiation in those without symptoms but with severe immune suppression [4].

Prior to 2013, the World Health Organization (WHO) recommended ART initiation in all HIV-infected individuals whose CD4 count has dropped to ≤350 cells/μl, irrespective of clinical symptoms. The WHO 2013 guidelines have raised the threshold for ART initiation to ≤500 cells/μl, with priority given to those with a CD4 count to ≤350 cells/μl, consistent with emerging data, indicating a clinical and public health benefit of earlier treatment and as part of a global effort to get 15 million HIV patients on ART by end of 2015 [5].

A number of technologies are available for CD4 enumeration, with considerable variation in cost, complexity, and operating requirements (Tables 1–5). The traditional approach to calculating absolute CD4+ T lymphocyte counts is to use the total leukocyte count (or lymphocyte count) obtained from the hematology analyzer and then use the percentage CD4+ T lymphocytes from the flow cytometric analysis to calculate the absolute values—the so-called “dual platform” (DP) approach. Quite often, however, two separate samples are used in the procedure, one to obtain the total leukocyte count using a hematology analyzer and one to undertake the flow cytometry, each having its own in-built variation. Thus, when the results from each are combined to determine the absolute CD4+ T lymphocyte count, the variation is compounded such that inter-laboratory variation between centers can be as high as 40%. Thus, the need to derive accurate and precise absolute CD4+ T lymphocyte counts has led to the development of instruments that can produce both percentage and absolute values, termed the”single platform” approach (SP).

**Table 1:** Operating characteristics of flow cytometric methods for CD4+ T-cell enumeration, using conventional flow cytometers.

FLOW CYTOMETRY USING CONVENTIONAL FLOW CYTOMETER^a^					
	Conventional dual platform	Bead-based single platform technology on conventional flow cytometer			Panleucogating
Assay name	Various	Trucount tubes	Flow-Count beads	Perfect-Count	PLG CD4
Manufacturer	Various	Becton Dickinson	Beckman Coulter	Cytognos	Beckman Coulter
		Compatible with various reagent systems and flow cytometers			Compatible with various reagent systems and flow cytometers
Instrumentation	Flow cytometer plus haematology analyser	Flow cytometer			Gating strategy
Assay principle	Absolute count calculated using results from flow cytometry together with the total lymphocyte count from haematology analyser	Absolute CD4 counts determined using ratio of CD4 to a known quantity of fluorescent beads—no need for haematology analyser			Gating strategy using total white cell count
		Trucount tubes contain premeasured quantity of lyophilised beads	Liquid beads need accurate pipetting by operator	Two different bead populations allow detection of inadequate mixing	
Parameters measured	Absolute CD4 and CD4%	Absolute CD4 and CD4%			Absolute CD4 and CD4%
	Others depend on reagent kits/methods				
Specimen	500μl whole blood in EDTA	Up to 100μl whole blood in EDTA			100μl whole blood in EDTA
Throughput	Up to 250 samples/day	Up to 250 samples/day			Up to 250 samples/day
Compatible with independent EQA/PT programmes?	Yes	Yes	Yes	Yes	Yes
Is there access to compatible full process QC reagent?	Yes	Yes	Yes	Yes	N/A
Maximum length of time from blood draw to testing	24 hours	24 hours			5 days
Can the test be performed on fixed/ stabilised blood	Yes	Yes	Yes	Yes	Yes
Complexity/training required	Complex. Significant training required.	Complex. Significant training required			Moderate training requirement
Environmental/energy issues	Requires uninterrupted mains electricity	Requires uninterrupted mains electricity			Requires uninterrupted mains electricity
Robustness to heat/humidity	Climate control recommended	Climate control recommended			Climate control recommended
	Reagents require refrigeration	Reagents require refrigeration			Reagents require refrigeration

^a^ Various flow cytometers available from various manufacturers. Examples include Becton Dickinson FACSCalibur, Beckman Coulter Epics XL.

**Table 2:** Operating characteristics of dedicated single platform CD4+ T-cell enumeration systems.

DEDICATED SINGLE PLATFORM CD4 SYSTEMS				
Assay name	FACSCount	Guava PCA	Partec CyFlow Counter or CyFLow SL_3	Apogee Auto40
Manufacturer	Becton Dickinson	Millipore (formerly Guava Technologies and now a division of Merck)	Partec	Apogee flow systems
Instrumentation	Flow cytometer	Flow cytometer	Flow cytometer	Flow cytometer
Assay principle	Dedicated single platform bead-based flow cytometer, two colour, no-lyse no-wash	Single platform volumetric flow cytometer. Microcapillary flow cell. Previously used EasyCD4 reagents (EasyCD4 system), since replaced by Auto CD4/CD4%. Company also produces the more complex easyCyte instruments, for research use only.	Volumetric SP flow cytometer. Up to 3 parameters (2 colour plus SSC). Simplest is single parameter CD4. For CD4%, 2 colour (CD45/4) + SSC. SL_3 (also called SL green) operates on the same principles but is adaptable to wider applications. Other CyFlow instruments such as SL blue have a wider range of parameters and potential applications.	Volumetric flow cytometer
Parameters measured	Absolute CD4 count and CD4%	Absolute CD4 count and CD4%	Absolute CD4 count and CD4%	Absolute CD4 and CD4%
Specimen	50μl whole blood in EDTA	10μl whole blood in EDTA	20μl whole blood in EDTA	50μl whole blood in EDTA
Throughput	≥30 samples/hour, at CD4 counts≥400	100–150 samples/day	250 samples/day, 400 samples/day with loader	20 samples/hour
Compatible with independent EQA/PT programmes?	Yes	Yes	Yes	No data available
Is there access to compatible full process QC reagent?	Yes	No manufacturer-produced full process control	Manufacturer produces dry stabilised control blood	No manufacturer-produced full process control
Maximum length of time from blood draw to testing	Stain within 48 hours of draw (24 hours for CD4%) (store at 20–25°C), analyse within 48 hours of staining.	48 hours	48 hours	Preferably run within 6–8 hours unless stabilised
Can the test be performed on fixed/ stabilised blood	Yes	Yes	Yes	Yes
Complexity/training required	One day training	One day training	Moderate training requirement	One day training
Environmental/energy issues	Requires uninterrupted mains electricity	Requires uninterrupted mains electricity Less biohazardous waste (reduced by 100 fold)	Mains electricity or car battery or solar panels	UPS with battery back up included. Optional inverter allows instrument to be powered by vehicle battery
Robustness to heat/humidity	10–35°C, 5–95% non-condensing humidity. Reagents require refrigeration.	Instrument: up to 35°C. 10–90% non-condensing relative humidity. Reagents require refrigeration.	Solid state laser more stable than water or air cooled gas lasers at high temperatures. Liquid reagents require refrigeration, or dry reagent kits available which can be stored at up to 60°C in dark for up to 12 months	Option of thermostable reagents with 9 month shelf life at room temperature. Instrument: 5–35°C, <90% humidity.

PCA = personal cell analysis, SSC = side scatter.

**Table 3:** Operating characteristics of manual technologies for CD4+ T-cell enumeration.

MANUAL METHODS		
Assay name	Coulter Manual CD4 Count Kit (Cytospheres)	Dynal T4 Quant Kit (Dynabeads)
Manufacturer	Beckman Coulter	Invitrogen/Dynal Biotech
Instrumentation	Light microscope, haemocytometer	Fluorescent or light microscope, haemocytometer, magnet
Assay principle	Inert latex spheres coated with monoclonal Ab form rosettes on contact with CD4 cells—readily visible by light microscopy	Magnetic polystyrene beads, coated with mouse monoclonal Ab, allow isolation of CD4+ T cells followed by counting using fluorescent (preferable) or light microscope
Parameters measured	Absolute CD4 count	Absolute CD4 count
Specimen	100μl whole blood in EDTA	125μl whole blood in EDTA or ACD
Throughput	10 samples/day. Operator fatigue a limitation. Suggest stop counting at 500 cells. Batch size limited to 2 samples	10 samples/day. Operator fatigue a limitation. Suggest stop counting at 500 cells. Batch size limited to 6 samples
Compatible with independent EQA/PT programmes?	No	No
Is there access to compatible full process QC reagent?	No	No
Maximum length of time from blood draw to testing	72 hours at 20°C	72 hours at 20°C
Can the test be performed on fixed/ stabilised blood	No	Compatible with Cyto-Chex (Streck, USA) stabilised refrigerated samples up to 9 days after collection. Not compatible with TransFix (CytoMark, UK).
Complexity/training required	1–3 days training	1–3 days training
Environmental/energy issues	Requires power for microscope	Requires power for microscope
Robustness to heat/humidity	Reagents need refrigeration	Reagents need refrigeration

**Table 4:** Operating characteristics of other commercially available technologies for CD4+ T-cell enumeration.

OTHER COMMERCIALLY AVAILABLE TECHNOLOGIES		
Assay name	PocH-100i/KX-21N with Dynal Dynabeads	CD4 Select
Manufacturer	Sysmex	i+MED Laboratories Co. Ltd.
Instrumentation	Haematology analyser	Haematology analyser, magnet, rotator
Assay principle	CD4 cell isolation using modified Dynal dynabeads, counting using haematology analyser with specific software	Ferrous beads coated with MT4 mAb bind to CD4 T cells, which are then removed using magnet. Whole blood and CD4-deplete blood counted on haematology analyser. CD4 count and % calculated using established formulae
Parameters measured	Absolute CD4 count and CD4%	Absolute CD4 and CD4%
Specimen	125 μl whole blood in EDTA	400μl whole blood in EDTA
Throughput	12 samples/hour	No data available
Compatible with independent EQA/PT programmes?	No EQA evaluation data available	No EQA evaluation data available
Is there access to compatible full process QC reagent?	No data available	No data available
Maximum length of time from blood draw to testing	24 hours at 4 or 20°C	No data available
Can the test be performed on fixed/ stabilised blood	No data available	No data available
Complexity/training required	Moderate complexity	Moderate complexity
Environmental/energy issues	Requires power for haematology analyser	Requires power for haematology analyser and rotator
Robustness to heat/humidity	No data available	No data available

**Table 5:** Operating characteristics of Point of Care (POC) technologies for CD4+ T-cell enumeration.

POINT OF CARE TECHNOLOGIES			
Assay name	PointCare NOW	Pima	CyFlow miniPOC
Manufacturer	PointCare Technologies	Alere (formerly Inverness Medical)	Partec
Instrumentation	Point of care instrument	Point of care instrument	Point of care instrument
Assay principle	CD4 and haematology contained in one unit.	All test reagents sealed within disposable cartridge.	Compact flow cytometric device. Uses dry reagents
	Gold nanoparticles conjugated to CD4 antibodies (no fluorescence). LED light source.	Portable analyser uses static image analysis and cell counting principles	
Parameters measured	Absolute CD4, CD4%, Hb, differential white cell count	Absolute CD4	Absolute CD4 and CD4%
Specimen	40μl whole blood in EDTA	25μl capillary or venous blood	20μl whole blood in EDTA
Throughput	50 samples/day	3 samples/hour	250 samples/day
Compatible with independent EQA/PT programmes?	No	Under investigation	Compatible with German EQA programme
Is there access to compatible full process QC reagent?	No data available	No data available	Company produce dry stabilised blood samples for QC
Maximum length of time from blood draw to testing	8 hours	24 hours	Provisional data suggest 24 hrs
Can the test be performed on fixed/ stabilised blood	No data available	Compatible with Transfix (CytoMark, UK)	No data available
Complexity/training required	Moderate	Minimal	Moderate
Environmental/energy issues	Requires mains or battery power	Requires mains or battery power	Requires mains or battery power
Robustness to heat/humidity	Reagents do not require refrigeration. Instrument: 18–34°C, <80% relative humidity, non-condensing	No refrigeration required (dried reagents)	No refrigeration required (dried reagents)

Two SP approaches are in widespread use today: volumetric and bead based. The principle of the volumetric approach is that a known volume of sample is passed through the flow cell (and interrogated by the laser beam) in a known amount of time. The alternative approach is to use bead-based technologies where a known number of beads is added to a known volume of sample thus allowing calculation of the bead to cell ratio and the subsequent calculation of the absolute cell count, in this instance, CD4+ T lymphocytes. An important feature of any absolute counting system is pipetting accuracy and minimum sample manipulation. The introduction of SP technologies has had a beneficial effect and lowered inter-laboratory variation in CD4 enumeration.

It is critical that country programmes consider whether these tests can give accurate and reproducible results as well as being appropriate for the setting [6]. In particular, we focussed on how bias and misclassification probabilities of different CD4 assays may affect eligibility for ART initiation. Misclassification probabilities give clinically useful measures of test performance and should be reported in evaluations of CD4 technologies. An upward misclassification around a treatment threshold means that a patient who should be eligible for treatment would be denied treatment, while a downward misclassification would not lead to ineligibility for treatment. To date, there have been no systematic reviews of the performance of CD4 technologies. Here we provide an evaluation of the performance characteristics of CD4 technologies through a systematic review of published literature.

## Methods

We performed a systematic review of studies evaluating the performance of CD4 enumeration technologies. A search of the Cochrane Library and the Centre for Reviews and Dissemination databases, including the Database of Abstracts of Reviews of Effects (DARE), the National Health Service Economic Evaluation Database (NHS EED) and the National Institute for Health Research Health Technology Assessment (NIHR HTA) database found no existing reviews addressing the review objective.

We followed standard guidance in performing the review [7]. Objectives and methods of the review were documented in a review protocol, which is included as S1 Methods.

### Eligibility criteria

Eligibility criteria were defined using the PICOS (Population, Interventions, Comparisons, Outcomes, Study Design) format. Studies evaluating the accuracy and/or precision of any CD4 technology commercially available at the time of the review were considered eligible for inclusion. Currently, no “gold” standard technology or internationally recognised reference preparation exists for CD4 enumeration, and a wide range of flow cytometric technologies have been used as comparators [6].

For the purposes of this review, we included studies that used as reference technologies any flow cytometric method considered to be acceptable by the WHO HIV diagnostics working group named in the review protocol (S1 Methods).

### Information Sources

Studies were identified by searching two electronic databases—MEDLINE and EMBASE, scanning the reference lists of the Nature supplement *Evaluating diagnostics*: *the CD4 guide*, and by inviting the WHO working group, whose members are authors of the Nature supplement to identify relevant studies for the review [6,8].

### Search Strategy

We used the following search terms to search the electronic databases: “CD4”, “technolog*”, “methodolog*”, “techn*”, “method*”, “test”, “evaluation”, “validation”, “accuracy”, “comparison”, “efficacy”, “performance”, “reproducibility”, “precision”, “flow AND cytometry”. Subject headings included: “CD4 antigen”, “antigens, CD4”, “CD4 lymphocyte count”, “CD4-positive T-lymphocytes”, “technology”, “methodology”, “technique”, “evaluation”, “clinical evaluation”, “economic evaluation”, “evaluation research”, “evaluation and follow up”, “instrument validation”, “validation process”, “validation study”, “evaluation studies as topic”, “validation studies”, and “flow cytometry”. Full electronic search strategies and review protocols are detailed in S1 Methods.

### Study selection

**Fig 1:**
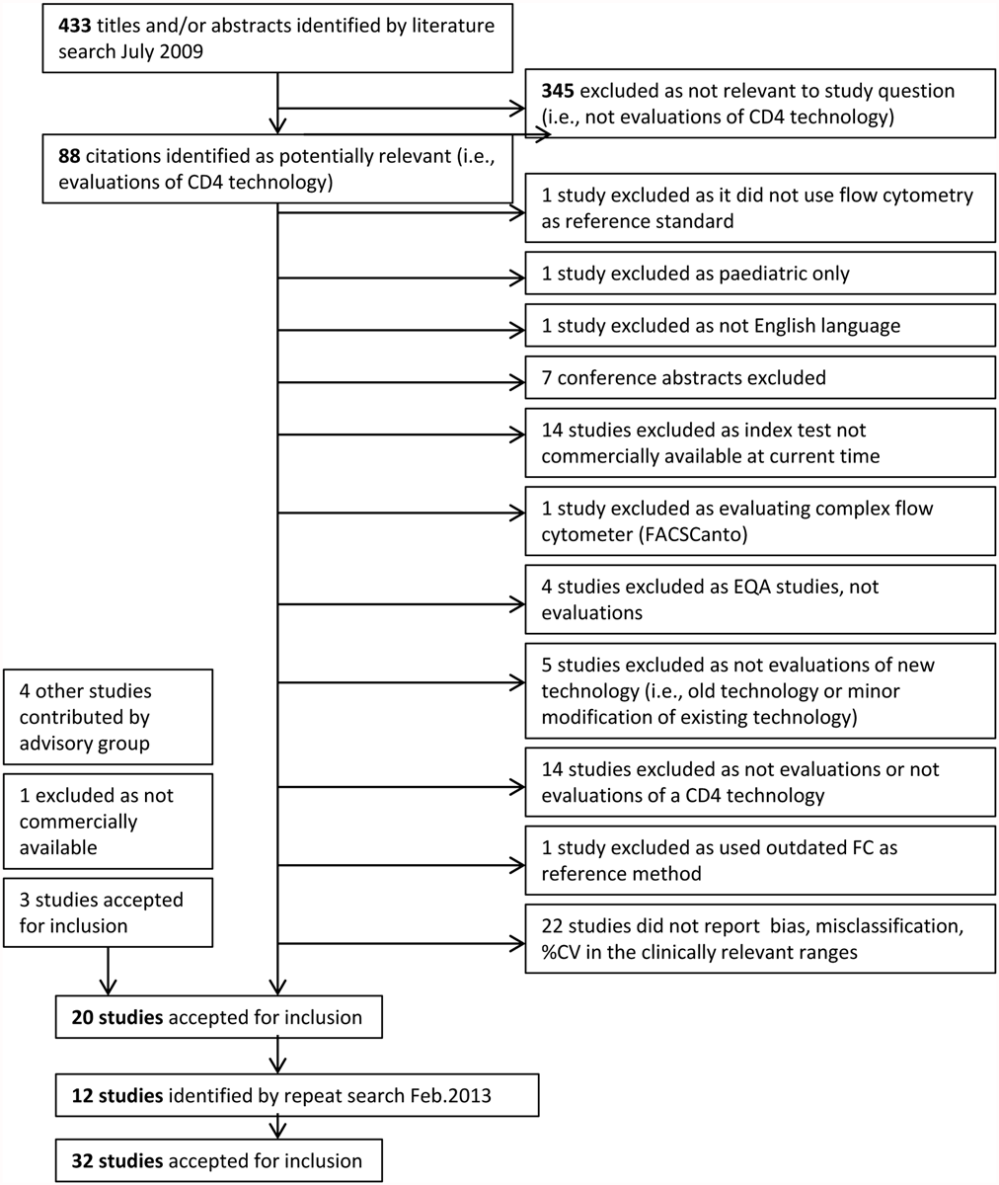
PRISMA flow diagram of study selection. EQA: external quality assurance, FC: flow cytometry.

Articles were exported from the search database to EndNote and screened for relevance (Fig. 1). Data were extracted by two independent reviewers (SG and KS) and disagreements resolved through consensus.

### Data extraction

The following data were extracted: study location, index test, reference test, and population (HIV positive or HIV positive and negative). Data on accuracy and precision included bias or mean difference and limits of agreement, misclassification probabilities (when sensitivity or specificity values were given, misclassification probabilities were calculated), and coefficient of variation. Where possible, HIV positive data alone were extracted. Where this was not possible, combined HIV positive and negative data were extracted. Only data within the clinically relevant range (thresholds of 200, 350, 500 cells/μl) were extracted as per the inclusion criteria.

Studies should report not only percent misclassification around clinically important CD4 cell thresholds (e.g., 200, 350 or 500 cells/μl), but should also report the magnitude of these misclassifications.

The secondary outcome measure addressed precision or reproducibility. Precision is particularly important when following a patient’s serial measurements using the same technology. Precision can be measured within-laboratory or between-laboratories and is expressed as percent coefficient of variation (%CV).

**Fig 2:**
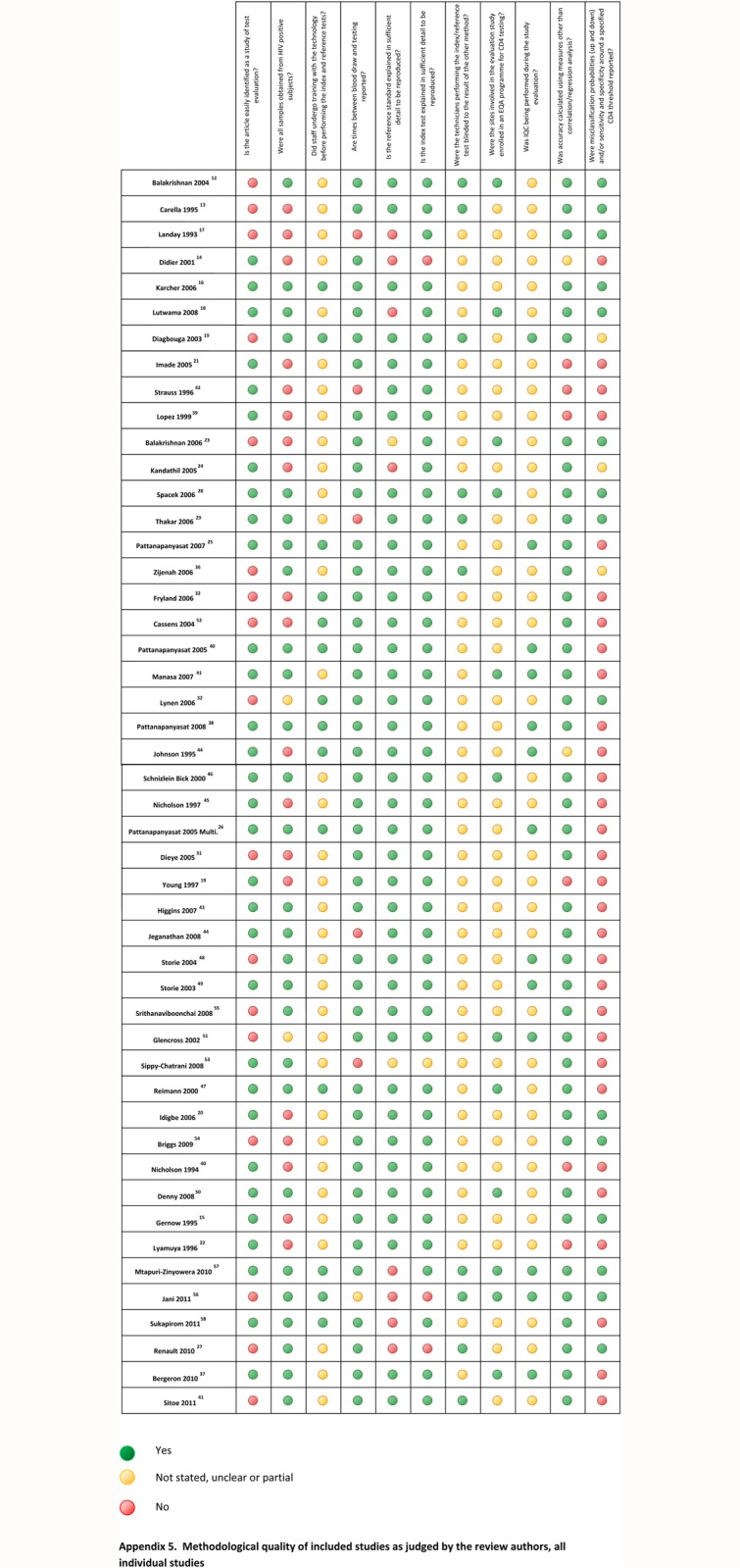
Methodological quality of included studies. EQA: external quality assurance, IQC: internal quality control.

Studies meeting inclusion criteria were also assessed for bias and quality on ten points drawn from the STARD guidelines (Fig. 2) [9]. This review has been reported following the PRISMA statement guidance for reporting of systematic reviews [10,11].

## Results

A summary of different commercially available CD4 technologies, including their assay principles, operational characteristics and compatibility with international external quality assurance programme reagents, is shown in Tables 1–5. Conventional flow cytometry based technologies include DP technologies such as the BD FACSCalibur (BD Biosciences, a division of Becton Dickinson, Franklin Lakes, NJ, USA [BD]) and the Epics XL (Beckman Coulter, Inc., Pasadena, CA, USA [BC]); bead-based SP technologies such as the BD Trucount Tube, the BC Flow Count Tubes, the Cytognos Perfect Count (Cytognos S.L., Salamanca, Spain [Cytognos]), and the BC Pan-leucogating (PLG) FlowCare CD4. Dedicated SP CD4 technologies include bead-based BD FACSCount, and three volumetric assays, Millipore Guava PCA (EMD Millipore, Darmstadt, Germany [Guava]), Partec CyFlow Counter (Partec, a division of Sysmex, Corporation, Kobe, Japan [Partec]) and Apogee Auto40 Flow Cytometer (Apogee Flow Systems, Hertfordshire, UK [Apogee]). There are two manual microscopy based counting methods, the BC Cytospheres and the Dynal T4 Quant (Dynal Biotech ASA, a division of Thermo Fisher Scientific Inc., Waltham, MA, USA [Dynal]). Point-of-care (POC) analysers include the Sysmex pocH-100i with Dynabeads from Dynal, the i+MED CD4 Select (i+ MED Laboratories, Bangkok, Thailand [i+ MED]), the Pima Analyzer (Alere Inc., Waltham, MA, USA [Alere]), and PointCare NOW (PointCare Technologies, Marlborough, MA, USA [PointCare]).

### Study selection

This systematic review was first performed in July 2009. Of the 433 studies in the search, 345 were excluded as they were not performance evaluations. After further triage, 20 studies that measured bias, misclassification and/or %CV were accepted for inclusion in this review (PRISMA flow diagram, Fig. 1). A second search was conducted in April 2013 using the same search strategy and review protocol with the goal of capturing more POC CD4 enumeration technologies in the review. An additional 12 studies were included. A summary of data extracted from all eligible studies with data on bias, misclassifications and/or %CV is shown in S1 Dataset.

### Study characteristics

A summary of study characteristics is shown in Table 6.

**Table 6:** Characteristics of included studies.

Study	Location	Laboratory or healthcare level at which the study was performed	Reference standard	Index test (test under evaluation)	Population	No. of samples used in accuracy studies (n)
**Balakrishnan 2004** ^**12**^	Chennai, India	Charitable foundation clinical/research lab	BD FACSCount	Coulter Cytospheres	HIV pos	122
**Carella 1995** ^**13**^	USA	Teaching hospital / research lab?	DP FC	Coulter Cytospheres	HIV pos and neg	117
**Landay 1993** ^**17**^	USA and Uganda	University research hospitals	DP FC	Coulter Cytospheres	HIV pos and neg	382
**Gernow 1995** ^**15**^	Denmark and Ivory Coast	Not clear	DP FC	Coulter Cytospheres	HIV pos and neg	123
**Didier 2001** ^**14**^	Paris	Teaching hospital lab	SP FC using BD Trucount tubes	Coulter Cytospheres	HIV pos and neg	55
			SP FC using BD Trucount tubes	Dynal Dynabeads	HIV pos and neg	46
			SP FC using BD Trucount tubes	BD FACSCount	HIV pos and neg	45
**Karcher 2006** ^**16**^	Uganda	Local lab, field conditions	DP FC	Coulter Cytospheres	HIV pos	131, from 102 donors
			DP FC	Partec CyFlow Counter CD4/SCC NLNW	HIV pos	128 from 121 donors
**Lutwama 2008** ^**18**^	Uganda	University laboratory	BD FACSCount or FACSCalibur	Coulter Cytospheres	HIV pos	1444 (≈497 donors)
			BD FACSCount or FACSCalibur	Dynal Dynabeads	HIV pos	1671
**Diagbouga 2003** ^**19**^	Five countries in West Africa	Local laboratories	SP FC using BD Trucount tubes	Dynal Dynabeads	HIV pos	657, from 301 donors
**Imade 2005** ^**21**^	Nigeria	University hospital laboratory	Partec CyFlow	Dynal Dynabeads	HIV pos and neg	40
**Idigbe 2006** ^**20**^	Nigeria	Research laboratory	BD FACSCount	Dynal Dynabeads	HIV pos and neg	97 (46 HIV pos, 51 neg)
**Balakrishnan 2006** ^**23**^	Chennai, India	Charitable foundation clinical/research lab	BD FACSCount	Guava Easy CD4	HIV pos and neg	228
**Kandathil 2005** ^**24**^	South India	Medical college laboratory and charitable foundation clinical/research laboratory	BD FACSCount	Guava Easy CD4	HIV pos and neg	72 (51 HIV pos, 21 neg)
**Spacek 2006** ^**28**^	United States	University laboratory	DP FC	Guava Easy CD4	HIV pos	77
	Uganda	University laboratory	BD FACSCount	Guava Easy CD4	HIV pos	141
**Thakar 2006** ^**29**^	Pune, India	Research institute	DP FC	Guava Easy CD4	HIV pos	79
**Pattanapanyasat 2007** ^**25**^	Bangkok, Thailand	Teaching hospital laboratory and ministry of public health-cdc lab	SP FC using BD Trucount tubes	Guava Easy CD4	HIV pos	250
			BD FACSCount	Guava Easy CD4	HIV pos	250
**Pattanapanyasat 2008** ^**26**^	Thailand	Teaching hospital laboratory	SP FC using BD Trucount tubes	Guava Easy CD4	HIV pos	150
			BD FACSCount	Guava Easy CD4	HIV pos	150
			SP FC using BD Trucount tubes	Partecv CyFlow SL_3	HIV pos	150
			BD FACSCount	Partec CyFlow SL_3	HIV pos	150
**Renault 2010** ^**27**^	Burkina Faso	Community laboratory	BD FACSCount	Guava Easy CD4	HIV pos	98
**Zijenah 2006** ^**36**^	Zimbabwe	University laboratories	BD FACSCount	Partec CyFlow Counter	HIV pos	150
**Fryland 2006** ^**32**^	Malawi	District hospital laboratory	BD FACSCount	Partec CyFlow Counter	HIV pos and neg	311 (276 HIV pos, 35 HIV neg)
**Cassens 2004** ^**30**^	Africa and Asia	‘Hospital laboratories’	Various	Partec CyFlow SL (SCC + CD4), NLNW	HIV pos and neg	434
**Pattanapanyasat 2005 ‘Evaluation of a new.’** ^**52**^	Bangkok, Thailand	Teaching hospital laboratory	SP FC using BD Trucount tubes	Partec CyFlow SL_3 (using single parameter CD4)	HIV pos	200
			BD FACSCount	Partec CyFlow SL_3 (using single parameter CD4)	HIV pos	200
**Manasa 2007** ^**34**^	Zimbabwe	National reference laboratory	DP FC	Partec CyFlow SL_3 (SCC +CD4 +CD45) (absolute CD4 and %CD4)	HIV pos	229
**Dieye 2005** ^**31**^	Senegal	Laboratory of a University Hospital	SP FC using BD Trucount tubes	Partec CyFlow SL Blue CD45/CD3/CD4 + SCCLNW	HIV pos and neg	121 (102 HIV pos donors, 28 neg)
			BD FACSCount	Partec CyFlow SL Blue CD45/CD3/CD4 + SCCNLNW		121 (102 HIV pos donors, 28 neg)
**Lynen 2006** ^**33**^	Cambodia	Laboratory of an NGO-run hospital	BD FACSCount	Partec CyFlow SL_3 CD4+SSC LNW manual gating	‘Likely to be HIV positive’	115
**Strauss 1996** ^**42**^	Belgium, UK, USA, Spain	Research laboratories, teaching hospital laboratory and manufacturer	DP FC	BD FACSCount	HIV pos and neg	
**Lopez 1999** ^**39**^	Spain and Portugal	7 centres: 6 hospital laboratories and one University laboratory	BD FACScan	BD FACSCount	HIV pos and neg	49 (37 HIV pos, 12 HIV neg)
**Johnson 1995** ^**38**^	USA	University laboratory?	DP FC	BD FACSCount	HIV pos and neg	47
**Nicholson 1994** ^**40**^	USA	Government laboratory	DP FC	BD FACSCount	Unknown for accuracy studies	50
**Lyamuya 1996** ^**22**^	Tanzania	Teaching hospital laboratory?	DP FC	BD FACSCount	HIV pos and neg	173
			DP FC	Dynal Dynabeads	HIV pos and neg	189
**Bergeron 2010** ^**37**^	Morocco/Canada	National reference laboratories	BD FACSCount	BD FACSCount using Rea T Count dry reagents	HIV pos	167
			BD FACSCalibur	BD FACSCount using Rea T Count dry reagents	HIV-pos	80
**Sitoe 2011** ^**41**^	Mozambique	Not stated	BD FACSCount using venous blood	BD FACSCount using capillary blood	HIV pos	101 adults
			BD FACSCalibur using venous blood	BD FACSCalibur using capillary blood	HIV pos	101 adults
**Schnizlein-Bick 2000** ^**46**^	USA	5 NIAID certified FC laboratories	DP FC	SP FC using BD Trucount tubes	HIV pos	60 common samples shipped to 5 sites, plus 14 local donors at each of 5 sites
**Nicholson 1997** ^**45**^	USA	University laboratory	DP FC	SP FC using BD Trucount tubes	HIV pos and neg	81
**Higgins 2007** ^**43**^	USA	Research laboratory	DP FC	SP FC using BD Trucount tubes	HIV pos	25
**Jeganathan 2008** ^**44**^	Australia	Central laboratory	DP FC	SP FC using BD Trucount tubes	HIV pos	60 paired samples from 18 patients
**Reimann 2000** ^**47**^	USA	5 clinical FC laboratories	DP FC	SP FC using BC Flow-Count fluorospheres: two colour + flow count	HIV pos	≥14 from each of 5 local sites = 71, plus 67 central specimens
			DP FC	SP FC using BC Flow-Count fluorospheres: tetraONE system	HIV pos	≥14 from each of 5 sites = 71, plus 71 central specimens
**Storie 2004** ^**48**^	UK	Teaching hospital laboratory?	SP FC using BD Trucount tubes	SP FC using Cytognos Perfect-Count beads	HIV pos	104
**Storie 2003** ^**49**^	UK	Teaching hospital laboratory?	SP FC with BD Trucount tubes	SP FC using flow rate calibration	HIV pos	113
			SP FC with BD Trucount tubes	DP PLG	HIV pos	113
**Denny 2008** ^**50**^	USA	5 FC laboratories	Varied by lab—SP or DP FC	DP PLG	HIV pos	99 samples sent to 5 labs, plus 58 local samples
**Pattanapanyasat 2005 ‘A multicentre evaluation.’** ^**35**^	Thailand	2xteaching hospital labs, 2xregional hospital labs	DP FC	DP PLG	HIV pos	611
**Glencross, 2002** ^**51**^	Johannesburg, South Africa	Teaching hospital laboratory	DP FC	DP PLG	Not stated	183
			Volumetric SP FC	DP PLG	Not stated	112
			SP FC using BD Trucount tubes	DP PLG	Not stated	112
**Sippy-Chatrani 2008** ^**53**^	Barbados	National HIV laboratory	SP FC with BC Flow-Count fluorospheres	SP PLG using BC FlowCARE	HIV pos	153
**Briggs 2009** ^**54**^	UK	laboratory	SP FC	Sysmex PocH-100i with Dynal Dynabeads	HIV positive for accuracy studies, HIV positive/ leukaemia /lymphoma for precision	115
**Srithanaviboonchai 2008** ^**55**^	Thailand	Research lab?	DP FC	i+MED CD4 select	HIV positive	100
**Mtapuri-Zinyowera 2010** ^**57**^	Zimbabwe	VCT clinic	SP FC using BD Trucount tubes	Alere Pima using capillary blood	HIV pos	165
**Jani 2011** ^**56**^	Mozambique	Primary healthcare clinics	BD FACSCalibur	Alere PIMA using capillary and venous blood	HIV pos	135
**Sukapirom 2011** ^**58**^	Thailand	Teaching hospital laboratory	BD FACSCount	Alere PIMA using venous blood	HIV pos	203
**Manabe 2012**	Uganda	Tertiary hospital laboratory	BD FACSCalibur with TruCount beads	Alere PIMA using capillary and venous blood	HIV pos	380
**Herbert 2013** ^**61**^	UK	Hospital laboratory (HIV service)	Coulter FC 500	Alere PIMA using capillary and venous blood	HIV pos	283
**Bergeron 2012** ^**60**^	Belgium, Canada, Mozambique, South Africa, USA	National laboratories	BD FACSCalibur, except in South Africa where the BC Epics-XL was used	PointCare NOW	HIV pos and neg	472
**Logan 2013** ^**62**^	USA	Research laboratory	BD FACSCalibur	mBio Snap Count using capillary and venous blood	HIV pos	146
**Mbopi-Keou 2012** ^**63**^	Cameroon	National Health Laboratory	BD FACSCalibur	Apogee Auto40 Flow Cytometer	HIV pos and neg	234 (adults and children)
**Mbopi-Keou 2012** ^**64**^	Cameroon	HIV reference Laboratory	BD FACSCalibur	Apogee Auto40 Flow Cytometer	volunteers	257 (adults and children)

FC = flow cytometry.

DP = dual platform.

SP = single platform.

SCC = side scatter.

BD = Becton Dickinson.

BC = Beckman Coulter.

LNW = lyse, no-wash.

NLNW = no-lyse, no-wash.

VCT = Voluntary Counselling and Testing.

At least one published performance evaluation study was found for each of the following technologies: BC Cytospheres [12–18], Dynal Dynabeads [14,18–22], Guava PCA [23–29], Partec CyFlow instruments [16,26,30–36], BD FACSCount [14,37–42]; BD Trucount tubes [43–46], BC Flow-Count fluorospheres [47], Cytognos Perfect-Count microspheres [48], SP Flow Cytometry using flow rate calibration [49–52]; BC PLG FlowCARE CD4 [53], Sysmex PocH-100i with Dynal Dynabeads [54], i+MED CD4 Select [55], Alere Pima Analyzer [56–58,61], PointCare NOW [60], Apogee Auto40 [59,63,64] and MBio Snap Count (MBio Diagnostics, Inc., Boulder, CO, USA [MBio]) [62].

No published, peer-reviewed performance evaluations of the commercially available technology Partec miniPOC were found by our literature search, and the MBio assay is not yet commercially available.

### Methodological quality of included studies

The findings of the quality assessment of included studies are summarised in Fig. 2. Most studies reported the index test (test under evaluation) and the reference standard in sufficient detail to be reproduced, but few studies reported whether staff at the evaluation sites were proficient at performing the reference standard and/or sufficiently trained on performing the index test. Few studies reported internal quality controls being performed during the evaluation period. Without these quality measures, it would be difficult to differentiate whether the bias or misclassification between the index and reference tests was due to differences in inherent test characteristics or to operator error.

Manufacturer involvement was evident in a number of studies. Seven studies declared one or more authors to be affiliated with the manufacturer of the index test [12,17,39,42,46,54]. Four studies were partially sponsored by the manufacturer [25,26,45,53]. One study stated that the manufacturer’s site was one of the study sites [42], and four studies declared donation of reagents or equipment by the manufacturer [15,29,38,40]. A further four studies could be considered to be calibration or test developers’ papers [30,49,51,55]. In the absence of definitions for a sponsored study versus an independent evaluation, it is not clear to what extent the inclusion of manufacturers as co-authors of papers influenced the study results.

### Accuracy

As there is no international standard for CD4 enumeration, a variety of reference standard technologies were used for evaluating the performance of new CD4 technologies, making it difficult to pool data on bias and misclassification across all studies.

### Bias

**Fig 3:**
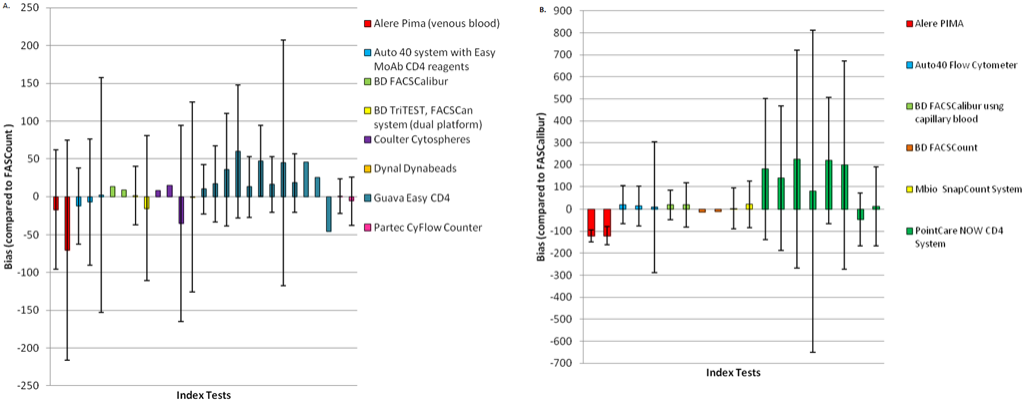
A. Bias compared to FASCount as the reference technology. B. Bias compared to FASCalibur as the reference technology.

Bias (mean difference) data were collated and represented graphically but only from studies that compared the index tests mean difference to the same reference technology (Table 6 and Fig. 3a, b). FACSCount and FACSCalibur were the most common reference technologies.

Bias was evaluated at CD4 thresholds of 200 cells/μl and 350 cells/μl using the BD FACSCount as a reference method. For the Pima Analyser, at CD4 counts at both <350 cells/μl and >350 cells/μl, the studies found that the Pima Analyser consistently underestimated CD4 counts by-16.6 cells/μl (limits of agreement-88.4, +55.3 cells/μl at <350 cells/μl) and at-70.7 cells/μl (limits of agreement-216.5, +75 cells/μl) [58]. However, when the Guava Easy CD4 was compared to the FACSCount, the bias for CD4 counts <350 cells/μl was +13 cells/μl (limits of agreement-27, +53) and for cell counts >350 cells/μl, bias was-45.3 cells/μl [23,27].

The FACSCalibur, in comparison to the FACSCount, overestimated CD4 counts by +13.1 cells/μl at values of <350 cells/μl [41]. However, bias estimation of manual bead based methods showed underestimation of CD4 counts at <350 cells/μl of-35.2 cells/μl (limits of agreement-164.9,+ 94.6) and -0.4 cells/μl (limits of agreement-126, +125.2) for BC Cytospheres and Dynal Dynabeads respectively [18].

When the overall bias for all CD4 technologies was calculated, this ranged from-70.7 to +47 cells/μl for CD4 counts >350 cells/ μl and -35.2 to +13.1 cells/μl for CD4 counts <350 cells/μl when compared to the FASCount as a reference method.

We then progressed to study bias data using a threshold of 200 cells/μl compared to FACSCount as a reference method. A total of six publications were identified covering the following technologies, the Apogee Auto 40 [59], Guava Easy CD4 [23,25–27], and Partec CyFlow Counter [26,52].

The four studies that had reported data at <200 cells/μl and compared the Guava Easy CD4 to the FACSCount, all had a positive bias that ranged from +10 to +45.5 cells/μl [23,25–27]. One study had data for CD4 counts >200 cells/μl and had a bias of +44.9 cells/μl (limits of agreement-112.6 to + 212.3) [25]. It is interesting to note that all studies reporting the performance of the Guava Easy CD4 showed that this assay overestimated CD4 counts compared to the FACSCount [23,25–27].

Two studies compared the Partec CyFlow Counter to the FACSCount. One showed an over-estimation by +0.8 cells/μl (limits of agreement-21.7 to +23.2) while the other showed an underestimation by-5.8 cells/μl (limits of agreement-37.6 to +25.9) [26,52].

Overall when comparing the technologies to the FACSCount as a reference method, bias ranged from +44.9 to -12.1 for CD4 counts >200 cells/ μl and +45.5 to -5.8 for CD4 counts <200 cells/ μl. Bias using FACSCalibur as a reference method showed similar results to that using FACSCount (Fig. 3a and b).

### Misclassification

**Fig 4:**
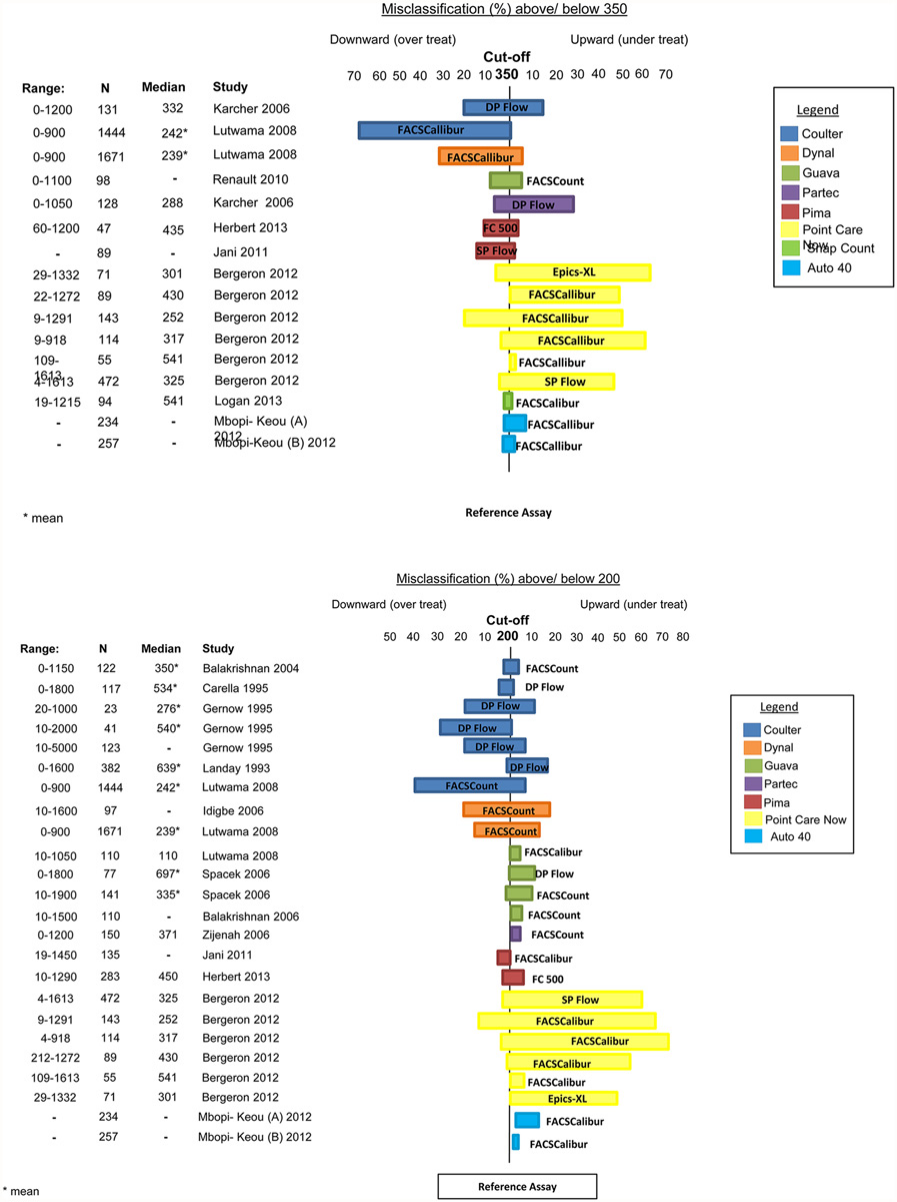
Misclassification (%), using a CD4 thresholds of 350 cells/μl and 200 cells/μl.

Fig. 4 showed the range of misclassifications at thresholds of 200 and 350 cells/μl of new CD4 assays compared to different reference standards.

Nine studies provided data on misclassification probabilities using a cut-off of 350 cells/μl [16,18,27,56,60–64]. Data were available on the following assays: BC Cytospheres [16,18], Dynal Dynabeads [18], Guava Easy CD4 [27], Partec CyFlow Counter [16], Pima Analyzer [56,61], PointCare NOW [60], MBio Snap Count [62], and Auto40 Flow Cytometer [63,64].

Two studies [63,64] evaluated the Auto40 Flow Cytometer compared to the FACSCalibur in Cameroon. One study [63] reported upward and downward misclassification probabilities of 8% and 2%, respectively, while another [64] found the likelihood of under-treatment (upward misclassification) to be 3% and the probability of over-treatment (downward misclassification) to be 2%.

Karcher *et al*. conducted a large trial in field conditions in Uganda comparing BC Cytospheres and the Partec CyFlow Counter to DP flow cytometry [16]. HIV positive patients were recruited, the majority of whom had CD4 counts within a range from 0 to1200 (median 332 cells/μl). Of samples with CD4 counts <350 cells/μl measured by DP flow cytometry, BC Cytospheres misclassified 16% of the patients as having CD4 counts of >350 cells/μl, thereby denying them of eligibility for treatment. Similarly for those with CD4 counts > 350 cells/μl measured by DP flow cytometry, BC Cytospheres misclassified 20%, indicating that 20% of patients not qualifying for treatment using the reference test would have done so if BC Cytospheres were used. Karcher *et al*. also compared the Partec CyFlow to the DP flow cytometer and found that of samples with counts <350 cells/μl, 29% were misclassified as having >350 cells/μl by the Partec CyFlow Counter, and 7% of samples with CD4 counts >350 cells/μl were misclassified downward as being <350 cells/μl [16].

Lutwama *et al*. conducted a large study of manual technologies in Uganda; they recruited only HIV positive patients, the majority of whom had CD4 counts within the clinically important range (range in study: 0–900 cells/μl) using the reference standard technology [18]. Of samples with counts of <350 cells/μl using the reference standard technology, BC Cytospheres misclassified only 1% as >350 cells/μl. However, of those with counts >350 cells/μl, 68% were misclassified as <350 cells/μl (indicating that 68% of patients not qualifying for treatment using the reference test would have done so if BC Cytospheres were used). Dynal Dynabeads had upward and downward misclassification probabilities of 6% and 30% respectively.

Renault *et al*. (2010) conducted a comparison study between the Guava Easy CD4 and FACSCount. Across a range of CD4 (0–1100 cells/μl), the upward and downward misclassification was calculated and found to be 6.1% and 9.4%, respectively [27].

One study reported on evaluations of the PointCare NOW assay (this instrument has since been re-marketed/rebranded as HumaCount CD4now (Human Diagnsotics Worldwide mbH, Weisbaden, Germany) in five countries (Mozambique, Belgium, Canada, USA and South Africa) [60]. Mozambique, Belgium, Canada and USA used the FACSCalibur as a reference standard while South Africa compared the PointCare NOW to the Epics XL. Upward and downward misclassification were reported by country: Mozambique, +51%, -20%; Belgium, +62%, -4%; Canada, +50%, -0%; USA, +0%, -3%; and for South Africa, +64%, -6%. Overall misclassification was also calculated, and it was found that testing with PointCare NOW would have led to 47% of patients with CD4 counts less than 350 cells/μl not eligible to receive treatment (upward misclassification) and 6% of patients with CD4 counts greater than 350 cells/μl eligible to receive treatment (downward misclassification).

Of the two studies evaluating the Pima Analyzer, Herbert *et al*. (2013) used the BC Cytomics FC 500 as a reference standard while Jani *et al*. compared the Pima Analyzer to the BD FACSCalibur [56,61]. Across the clinically relevant range (60–1200 cells/μl), Herbert *et al*. found the upward and downward misclassification to be 6.1% and 9.4% respectively. MBio Snap Count was evaluated by Logan *et al*. (2013) and compared to the FACSCalibur [62]. Of the 94 samples, 2.1% were misclassified upward and 3.2% were misclassified downward at a threshold of 350 cells/μl.

Thirteen studies presented misclassification data using a CD4 cut-off of 200 cells/μl; these are presented in Fig. 4b [12,13,15,17,18,20,23,28,36,56,60,61,63,64].

Even through misclassification probabilities can be influenced by the number of patients with CD4 counts close to the threshold in each study, Pointcare NOW showed an overall tendancy towards upward misclassification at both thresholds of 200 and 350 cells/μl. Most other technologies showed misclassification probabilities of <10%.

### Precision

**Fig 5:**
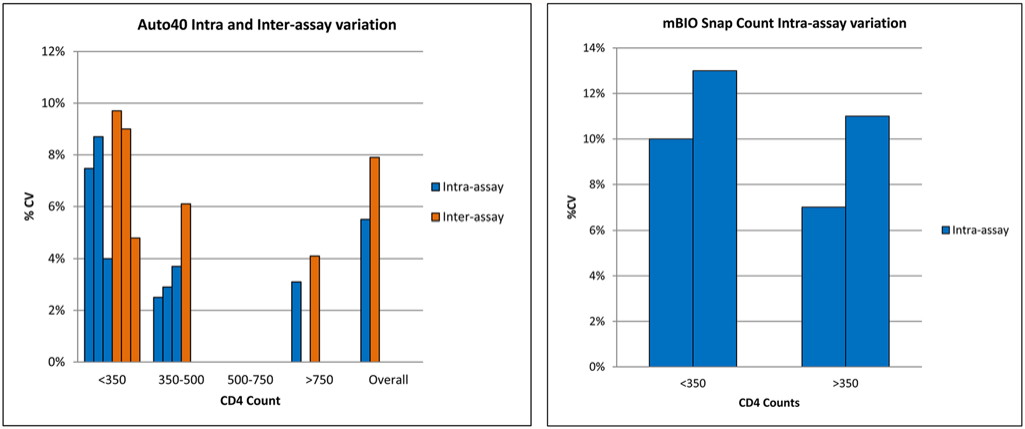
Intra- and inter-assay variation (% CV) for the Apogee Auto 40 and for the MBio SnapCount at thresholds <350 cells/mL and >350 cells/mL.

Forty-four percent of studies reported within-laboratory precision of absolute CD4 count measurement, using replicates of fresh whole blood^.^ [15,17,19,21,24,29,32–34,38–42,45–47,50,54,56,58] Fig. 5 shows the inter- and intra assay variations expressed as % CV for the Apogee Auto assay and the intra-assay variation for the mBio SnapCount [62,63].

Five studies reported between-laboratory precision using whole blood. Two were studies evaluating BD FACSCount, [39,42] one evaluated Panleucogating, [50] and two were studies evaluating bead-based SP technology (BD Trucount tubes and BC Flow-Count fluorospheres) [46,47]. Gernow *et al*. studied the reproducibility of BC Cytospheres and found poor precision, with a coefficient of variation of 58% [15]. The study by Landay et al., however, found precision levels more in keeping with the other technologies [17].

Overall, in studies addressing between-laboratory precision, SP flow cytometry using BD Trucount tubes or BC Flow-Count fluorospheres showed less inter-laboratory variability than the DP comparators. [46,47] In addition, Denny *et al*. demonstrated improved inter-laboratory precision using DP Panleucogating compared with technologies which included DP or SP conventional flow cytometry [50]. External quality assurance data showed that the BD FACSCount, which is most commonly used a reference standard for CD4 assay evaluations, has within-laboratory and between-laboratory precision of 15% or less [38,39,42,46].

## Discussion

This review highlights the difficulties of answering clinically relevant questions about CD4 test performance from the published literature. A minority of studies reported clinically useful measures of accuracy, and few POC tests were carried out under field conditions.

It can be seen that whatever technology is chosen, there is variability associated with CD4 measurement. It should be noted that there is also significant physiological variability in CD4 count, that may account for as much as, if not more than, technical variability of CD4 measurement [65–67]. Performance characteristics vary between technologies and for the same technology depending on the reference technology used as a comparator. These characteristics have important implications both for individual patient management and for HIV treatment programmes. It is essential to consider assay performance as well as operating characteristics when choosing a technology. However, these data are not always available in the literature, and currently, evaluations are not sufficiently robust or comprehensive to give a clear idea of the comparative merit of different technologies.

Misclassification probabilities describe the likelihood that a test will incorrectly categorise a result as higher or lower than a given cut-off value as measured by a reference standard. They are clinically relevant measures of accuracy, as they can be used to assess the likelihood that a patient will be incorrectly classified above or below a defined CD4 threshold used in clinical decision making. Misclassification probabilities for the same assay can vary not only because the test is compared to different reference standards, but also because the probabilities are affected by the number of samples clustered around the thresholds of 200 or 350 cells/ul.

Two types of misclassification can be defined—upward misclassification and downward misclassification. Upward misclassification around a treatment threshold may be the most clinically important, leading to a delay in starting ART in some patients, with potentially harmful consequences. Downward misclassification on the other hand would be expected to lead to ART use earlier than indicated, with potential implications for cost and drug exposure. Given the trend towards earlier initiation in global and national guidelines, a degree of over-treatment is likely to be preferred over significant under-treatment [68]. Furthermore, the use of CD4 counts alone to assess ART immunological failure in the absence of viral load monitoring will, because of the biases observed, lead to some patients not receiving the appropriate clinical intervention.

Misclassification data showed that manual technologies [18], particularly the method using BC Cytospheres, were associated with substantial downward misclassification. It would therefore be expected that the implementation of these tests would lead to the decision to treat potentially large numbers of additional patients who have CD4 counts above the guideline threshold when using the reference test. Less upward misclassification was seen, suggesting that under-treatment might be less of a problem with these technologies. Upward misclassification by either manual technology is however likely to be an underestimate as the majority of counts in this study were very low (less than 25% of samples had counts >200 cells/μl); if the tests were to be used in a population with counts closer to the treatment threshold (as might be the case if used primarily in asymptomatic patients), upwards misclassification would be expected to be higher.

Limited misclassification data were available for the Partec CyFlow instruments. Of concern, one study evaluating the Partec CyFlow Counter under field conditions found 29% upward misclassification; that is, 29% of patients potentially eligible for treatment may have been denied treatment if the Partec CyFlow Counter-determined CD4 counts were the only criteria for assessing eligibility [16]. No other studies of the Partec CyFlow Counter or other CyFlow instruments reported misclassification probabilities at 350 cells/μl. Therefore, we do not know if this finding was replicated elsewhere. The Guava PCA (using EasyCD4 reagents) and the Pima Analyzer showed acceptable upward and downward misclassification rates.

There is some disparity in precision reported for the BC Cytospheres, and the reason for this disparity is not clear. The CD4 counts of the 19 samples used for replicate analysis in the study conducted by Gernow *et al* were not stated; however, they included 12 HIV negative samples that might be assumed to have high counts [15]. Given that several papers found BC Cytospheres to have poorer performance at higher counts, this may be in keeping with poor reproducibility in these replicates. Another study, conducted by Landay *et al*., found better precision on a sample with a CD4 count of 1200 (%CV 3·5%) than on a sample with a CD4 count of 200 (%CV 10·8%) [17]. Manual methods, although employing simple technology, are labor intensive and require significant user skill. Inadequate training, lack of supervision, or user fatigue may lead to poor performance of these techniques. Neither study described the training received by technicians performing the manual tests, nor reported blinding. Between-laboratory precision is likely to be superior with SP technologies (using BD Trucount tubes or BC Flow-Count fluorospheres) than with conventional DP technologies. As more point-of-care devices are introduced to lower levels of the health system, where training and supervision can be challenging, the lack of adequate training and supervision may introduce additional sources of error, contributing to decreased assay precision. This should be addressed through the development of a comprehensive training and supervision policy and implementation plan for the introduction of POC devices. In addition to the studies presented in this review, evaluations have been performed by government agencies and other national bodies that have not been published in peer-reviewed journals. An evaluation of BC Cytospheres published by the Medical Devices Agency of the UK included samples from 17 HIV positive subjects, and compared BC Cytospheres against DP flow cytometry [69]. Accuracy was reported using assessment of bias; misclassification probabilities were not reported. Imprecision was addressed using 6–7 replicates of 6 samples, and found a CV range of 3·2–17·6% (mean 8·5%). Unpublished evaluations have not been included in this review.

A recent review of external quality assurance (EQA) programmes involving 58,626 CD4 data sets from over 3,000 laboratories over a 12-year period show that SP technologies consistently give lower relative errors and confidence limits than DP technologies at clinically significant absolute CD4 counts [70].

### Limitations of this review

Limitations include the fact that we only included papers published in the English language, and we may have overlooked data because of this limitation. Limiting the search to the peer-reviewed literature may have overlooked robust evaluations conducted by national reference facilities or similar institutions.

It is important to consider that the reference standard technologies themselves are not perfect. Misclassification assumes that the reference result is accurate, i.e., the closest approximation to the truth. Thus, a result considered as a misclassification may in fact be correct. The reference technology if performed once may give a result of 340cells/ul, but if performed in duplicate using the same specimen may give results of 340 and 360. It may be important for the reference technology to be performed in duplicate and only when a concordant result is obtained around a threshold of 350 can it be used as the reference standard for the new test.

What constitutes an “acceptable” margin of error and misclassification probabilities around a threshold remain undefined and may vary among sites, depending on local factors such as the distribution of CD4 counts among asymptomatic patients, how often patients undergo repeat CD4 testing, and the implications of potential overtreatment (e.g., cost, long term risk of drug toxicity). However, given the move towards earlier treatment and the use of better-tolerated, less toxic drugs, misclassification that results in overtreatment may be more acceptable than would have previously been the case. It is relatively straightforward for national programmes to decide which technology best fits their needs based purely on cost and operating characteristics; it may be harder to decide what performance characteristics are acceptable, and harder still to obtain data on test performance to inform choice.

Given the potential for testing error, laboratory participation in EQA programmes and access to quality control (QC) reagents are essential. EQA information is not mentioned in the publications included in the review. Without this information, the proficiency of the laboratory staff performing the testing may have contributed to the errors and variation in addition to the assays themselves.

## Conclusions

A wide range of bias and percent misclassification around treatment thresholds over the clinically relevant range were reported on the CD4 enumeration technologies included in this review. Less than half the studies reporting assay precision or reproducibility of the CD4 values obtained. This is a rapidly evolving field with new tests under development, and with existing instruments and reagents being regularly replaced by updated versions. A systematic review of POC tests compared to laboratory-based technologies showed that POC CD4 testing can increase retention in care prior to treatment initiation and can also reduce time to eligibility assessment resulting in more eligible patients being initiated on life-saving treatment [71]. The lack of standardized methodology on test evaluation, including consensus on reference standards, is a barrier to assessing relative assay performance and could hinder the introduction of new POC assays in countries where they are most needed.

## Supporting Information

S1 Methods Systematic review protocol. (DOC)

S1 Dataset Summary of data extracted from eligible studies. (XLS)

S1 PRISMA Checklist (DOC)
