# Supplementary material for: CD4 Enumeration Technologies: A Systematic Review of Test Performance for Determining Eligibility for Antiretroviral Therapy
Source: PLoS One. 2015 Mar 19;10(3):e0115019. doi: 10.1371/journal.pone.0115019 (PMC4366094; doi:10.1371/journal.pone.0115019)
Supplement: S1 Methods — (DOC) [file pone.0115019.s001.doc]

**Supporting Information S1**

**Review protocol**

**A systematic review of studies evaluating the performance of CD4 enumeration technologies**

London School of Hygiene and Tropical Medicine for the World Health Organisation

**Review Team**

Sarah Glover, Kimberly Sollis, Rosanna Peeling

**Advisory Group**

Suzanne M Crowe, Burnet Institute and Monash University, Melbourne, Australia

Ben Cheng, Pangaea Global AIDS Foundation, Oakland, USA

David Barnett, UK NEQAS for Leucocyte Immunophenotyping, Sheffield, England

Thomas J Spira, Centers for Disease Control and Prevention, Atlanta, GA, USA

Wendy S. Stevens, University of the Witwatersrand and National Health Laboratory Services, South Africa

**Background**

HIV has particular tropism for CD4 cells and destruction of CD4 bearing cells mediates many of the harms HIV causes. Measurement of CD4 bearing T lymphocytes in peripheral blood is therefore a critical laboratory assay for evaluation and monitoring of patients with HIV. It is used to determine the immunological stage of HIV infection, for identifying when to start antiretroviral therapy (ART), to identify those most at risk of developing immune reconstitution syndrome, for evaluating the response to treatment, and for recognizing failure of treatment, and to identify women with HIV who are more likely to transmit HIV to their infants.

WHO therefore encourages national programs to increase access to CD4 measurement technologies to improve the quality of HIV prevention, care and treatment.

A range of techniques for determining CD4 exist, however the range of cost, feasibility, reliability, reproducibility and ease of use vary considerably. In general the cost and complexity mean that access to CD4 and viral load measurement is still highly restricted in many low income countries in Africa and Asia. The infrastructure of clinical laboratories located in many developing countries is often inappropriate to support routine determination of CD4 and viral load and high costs of equipment, reagents and problems associated with its maintenance further restrict access.

Simpler and less expensive methods for enumeration of CD4+ T lymphocytes have been explored and are likely to now become available. The introduction of such techniques and assays should allow the follow-up of increasing numbers of patients in sites that are not fully equipped to perform the full range of laboratory investigations and may allow CD4 testing distant from major cities in developing countries.

**Review objectives**

To assess the performance of available CD4 enumeration technologies

**Methodology**

A search of the Cochrane Library and the Centre for Reviews and Dissemination databases (including DARE, NHS EED and HTA) found no existing reviews addressing the review objective

**Eligibility criteria**

**PICOS**

**Population**

Study population must include (but not necessarily be limited to) HIV infected adults, in any geographical location

**Interventions (index tests or tests under evaluation)**

Any technology used for the enumeration of CD4 count, including (but not limited to) manual bead-based technologies, flow cytometry systems requiring minimal technical input, and flow cytometry systems requiring significant operator input or flow cytometry skill

**Reference standard/comparators**

Include studies using any acceptable (currently or at the time of the study) flow cytometric method as the reference standard

**Outcomes**

Include studies evaluating *accuracy* (including eg bias and/or misclassification probabilities) and/or *precision.*

Include studies calculating *sensitivity, specificity, PPV and NPV* for a defined cut off CD4 value eg 200

**Study design**

Include evaluation studies using an acceptable reference technology

**Identifying research evidence**

**Search strategy**

Databases: MEDLINE and EMBASE

Reference list of Nature supplement: Evaluating diagnostics: the CD4 guide. Nature Review Microbiology November 2008 Vol 6 No 11

Contacting experts (advisory group)

**Search terms**

Search terms to include: “CD4”, “technolog*”, “methodolog*”, “techn*”, “method*”, “test”, “evaluation”, “validation”, “accuracy”, “comparison”, “efficacy”, “performance”, “reproducibility”, “precision”, “flow AND cytometry”

Subject headings to include “CD4 antigen”, “Antigens, CD4”, “CD4 lymphocyte count”, “CD4-positive T-lymphocytes”, “technology”, “methodology”, “technique”, “evaluation”, “clinical evaluation”, “economic evaluation”, “evaluation research”, “evaluation and follow up”, “instrument validation”, “validation process”, “validation study”, “evaluation studies as topic”, “validation studies”, “flow cytometry”

**Date**

1990-March 2013

**Language**

English only

**Publication type/status**

Only published work in peer reviewed journals

**Study selection**

Stage 1: Screening of titles/abstracts against inclusion criteria.

Titles and abstracts where available will be screened and either accepted, or rejected as not relevant, or rejected due to failure to meet inclusion criteria – if so, the reason will be specified

Stage 2: Full papers obtained and assessed against inclusion criteria, if rejected due to failure to meet inclusion criteria, the reason will be specified

Titles +/- abstracts reviewed for relevance

Articles identified as potentially relevant (i.e. evaluations of CD4 enumeration technology)

Excluded as not relevant to study question i.e. not evaluation of CD4 technology

Studies excluded due to failure to meet inclusion criteria

Studies excluded due to failure to meet inclusion criteria

Studies excluded due to failure to meet inclusion criteria

Studies excluded due to failure to meet inclusion criteria

Articles accepted for inclusion

**Data extraction form**

| **Study** |  |
| --- | --- |
| **Location (country)** |  |
| **Location (reference laboratory or local laboratory level?)** |  |
| **Index test** |  |
| **Reference standard** |  |
| **Population (HIV status only)** |  |
| **Range of CD4^#^ or subgroup** |  |
| **No of samples (n)** |  |
| **Accuracy measurements:** | |
| **Bias (cells/µl)^*^** |  |
| **Limits of agreement (cells/µl)** |  |
| **Maximum observed difference†** |  |
| **% Misclassification up (CD4 cut off, cells/µl)** |  |
| **% Misclassification down (CD4 cut off, cells/µl)** |  |
| **Precision measurements:** | |
| **No of replicates per donor, no of donors** |  |
| **CD4 count of replicate sample (cells/µl)** |  |
| **%CV, index test, reference test** |  |

Where HIV positive data can be extracted, this alone will be included. When this is not possible, combined HIVpos/neg data included.

#Range of CD4 (read from plot if not stated in text), mean, SD (all as measured by reference test, unless only Bland-Altman plot available for estimating range, in which case as measured by mean of two tests) (cells/µl)

*mean difference unless stated otherwise

†read from plot if not stated in text

**Quality assessment**

| **Title/abstract** | Is the article easily identified as an evaluation of CD4 technology? |  |
| --- | --- | --- |
| **Methods** |  |  |
| Population | Were all samples obtained from HIV positive subjects? |  |
| Test methods | Is the training received by the technicians prior to performing the index and reference tests reported? |  |
|  | Is the time between blood draw and testing reported? |  |
|  | Is the reference standard explained in sufficient detail to be reproduced? |  |
|  | Is the index test explained in sufficient detail to be reproduced? |  |
|  | Were the technicians performing the index/reference test blinded to the result of the other method? |  |
|  | Were the sites involved in the evaluation study enrolled in an External Quality Assurance (EQA) programme for CD4 testing? |  |
|  | Was Internal Quality Control (IQC) being performed during the study evaluation? |  |
| Statistical methods | Did the study use measures other than correlation analysis to report accuracy? |  |
|  | Did the study report misclassification probabilities and/or sensitivity and specificity around a clinically relevant CD4 value? |  |

**Data synthesis**

Extracted data will be collated and summarized. If sufficient appropriate data are available, meta-analysis may be undertaken.

**Dissemination**

Findings of the systematic review will be presented as a report to the World Health Organization, and published in a peer-reviewed journal.
